# Use of Commercially Available Large Language Models to Generate Information Leaflets on Post–Intensive Care Syndrome: Clinical Utility Assessment

**DOI:** 10.2196/81606

**Published:** 2026-05-14

**Authors:** Nanami Hata, Takehiko Oami, Eiryo Kawakami, Akiko Hanai, Taka-aki Nakada

**Affiliations:** 1 Department of Emergency and Critical Care Medicine, Chiba University Graduate School of Medicine, 1-8-1 Inohana, Chuo, Chiba, 260-8677, Japan, +81-43-226-2372; 2 Department of Artificial Intelligence Medicine, Graduate School of Medicine, Chiba University, Chiba, Japan; 3 Institute for Advanced Academic Research, Chiba University, Chiba, Japan; 4 Predictive Medicine Special Project (PMSP), RIKEN Center for Integrative Medical Sciences (IMS), RIKEN, Kanagawa, Japan; 5 Division of Applied Mathematical Science, RIKEN Center for Interdisciplinary Theoretical and Mathematical Sciences, RIKEN, Kanagawa, Japan; 6 Faculty of Informatics, Graduate School of Informatics, Chiba University, Chiba, Japan

**Keywords:** large language models, post–intensive care syndrome, clinical practice guidelines, artificial intelligence, generative AI, retrieval-augmented generation

## Abstract

**Background:**

Patients and their families without medical knowledge may find professional health care information difficult to understand. The use of large language models (LLMs) to simplify and translate complex medical content holds promise for improving comprehension while reducing the burden on health care providers tasked with delivering explanations.

**Objective:**

This study aims to evaluate the quality of information leaflets generated using commercially available LLMs.

**Methods:**

Informational texts on post–intensive care syndrome were generated using 6 different LLMs and 4 prompt designs with varying levels of instructional guidance. Clinical practice guideline documents were uploaded and provided to the models as reference context, reflecting a pragmatic clinical scenario without model tuning or advanced retrieval pipelines. In total, 72 texts were generated (6 models × 4 prompts × 3 outputs). After excluding texts shorter than 500 characters (n=16) and those without explicit mention of post–intensive care syndrome (n=3), 53 texts remained. To enable balanced human evaluation across model-prompt combinations, the longest eligible response from each pair was selected (4 prompts × 4 models; n=16). Following independent expert review by 2 medical specialists, 7 texts were excluded, leaving 9 texts for final analysis. Ten individuals, including health care professionals and nonmedical personnel, assessed the texts using a 10-point Likert scale across multiple quality domains. An LLM-based parallel assessment was also conducted, and scores were compared across models and evaluator groups.

**Results:**

In the human evaluation of the selected 9 texts, the generated texts achieved an average score of 6.8 or higher across all evaluation criteria, with no potentially harmful content identified. The text generated by LLaMA 3 70B, using a step-by-step approach combined with text-augmented prompting based on clinical guidelines, received the highest overall score, whereas the lowest-rated text was produced using a simple prompt without text augmentation. Although no consistent trends were observed across LLMs or prompt engineering strategies, text-augmented prompting was generally associated with higher evaluation scores. Ratings differed between professional and nonprofessional evaluators. Given the feasibility-driven screening process and the resulting limited sample size, the findings should be interpreted as exploratory and descriptive rather than definitive estimates of overall model performance.

**Conclusions:**

Among the selected texts included in the final human evaluation, informational materials generated using commercially available LLMs were generally rated as acceptable by human evaluators, and none contained harmful content. These findings suggest that LLMs may support the development of patient-facing informational materials under feasibility-constrained conditions, although further validation with larger and more diverse samples is warranted.

## Introduction

Medical documents such as clinical practice guidelines are intended to support decision-making by health care professionals and patients based on recommendations for standard examinations and treatments [1,2]. However, because these guidelines are primarily written by medical specialists, their content is typically challenging for patients and their families to comprehend. Moreover, adapting these documents for diverse audiences is time-consuming and labor-intensive. A study evaluating the costs associated with creating patient education materials found that the average annual expenditure per center was approximately $65,401 for pamphlet development and $19,819 for annual review, highlighting the significant investment required for these endeavors [3].

With recent advancements in generative artificial intelligence (AI), large language models (LLMs) have become capable of instantly summarizing documents and rephrasing content in specific contexts [4-6]. Leveraging this technology to tailor professional medical content for patients and their families may enhance the readability of materials originally designed for health care providers [7,8]. Although the use of commercially available LLMs renders medical documents more patient-friendly, hallucinations can result in content inaccuracy that can be challenging to regulate [9]. LLMs can be augmented with domain-specific knowledge through retrieval-augmented generation [10], allowing the integration of up-to-date and contextually relevant information into generated outputs [11]. The creation of disease- or condition-specific informational leaflets through LLMs enhances patient and family comprehension and reduces the burden on clinicians. However, the feasibility and validity of using commercially available LLMs to generate information leaflets for different audiences remain inadequately explored.

Post–intensive care syndrome (PICS) encompasses physical, cognitive, and psychological impairments that occur during or after an intensive care unit stay and frequently persist after hospital discharge [12]. PICS affects the long-term prognosis and quality of life of intensive care unit survivors and has a significant psychological impact on their families [13]. A growing concern in emergency and critical care medicine is promoting the awareness of PICS among patients and their families, which is essential for its effective prevention and management [14]. The focus on PICS underscores a critical unmet medical need. Patients, their families, and health care professionals are often required to respond to this condition unexpectedly without access to adequate informational resources. The development of condition-specific, readable, and medically accurate informational leaflets through LLMs and text-augmented prompting approach based on clinical guidelines has the potential to improve comprehension among nonexpert audiences and reduce the communication burden on clinicians during acute and emotionally charged periods [15].

This study aimed to evaluate the clinical utility and safety of information leaflets on PICS for patients and their families, developed using an accessible commercially available LLM. For the qualitative assessment, medical experts, nonexpert individuals, and an LLM were used to review the generated content.

## Methods

### Study Design and Settings

An evaluation study was conducted to evaluate the interpretability and readability of information leaflets on PICS generated using LLMs. Using prompt engineering and text-augmented prompting approach, we generated patient-friendly explanations of clinical practice guidelines and evaluated the quality of the generated materials.

### Prompt Design and Content Generation

Four prompts were designed to explore different content generation strategies and levels of guidance (Multimedia Appendix 1).

- Prompt 1: zero-shot without the uploaded context—Simple instructions directed the model to explain PICS in a patient-friendly manner without the use of examples or an external context.
- Prompt 2: zero-shot with the uploaded context—Basic instructions to explain PICS, supplemented with clinical practice guideline content, were provided as an additional context via the text-augmented prompting approach.
- Prompt 3: few-shot with the uploaded context—The model was provided with specific examples of questions and answers to guide responses. The text-augmented prompting approach was used to ensure evidence-based content.
- Prompt 4: Step-by-step with the uploaded context—A detailed instruction set guided the model to retrieve relevant information and deliver a comprehensive explanation of PICS, including causes, symptoms, treatments, and practical advice for patients and families. Although prompt 4 instructed the model to “search for the latest clinical guidelines,” the models operated without internet access. This instruction was intended as a directive for contextual grounding—specifically, to command the model to treat the uploaded document as the “latest” source retrieved from a search, thereby prioritizing the provided context over the model’s pretrained internal knowledge.

These prompts were applied to 6 LLMs—ChatGPT-4o, LLaMA 3 70B, MedItron:7b, Gemma, Mistral, and MedLLaMA2—with 3 outputs generated per prompt-model combination, resulting in a total of 72 texts (Multimedia Appendix 2). To reflect the models most likely to be used by health care professionals, we focused on available models through Ollama and selected the 4 models with the highest number of pulls as of May 2024, as well as the 2 models with the highest number of pulls identified by searching for the keyword “medical.” For open-source models, we used Ollama with its default generation settings at the time of execution (July 2024) without parameter tuning. The settings included a temperature of 0.8, repeat penalty of 1.1, repeat_last_n of 64, seed of 0, and a context window size (num_ctx) of 2048 tokens. We did not perform token counting or explicit verification of context retention during generation. Therefore, depending on the combined length of the system instructions, prompts, uploaded guideline text, and generated output, partial truncation of the contextual input may have occurred. This configuration was maintained to simulate a standard user environment where context window adjustments are not typically performed.

For prompt conditions using guideline context (prompts 2‐4), the generation workflow was as follows:

1. The PICS section of the Japanese version of the Surviving Sepsis Campaign Guidelines was prepared as an uploaded reference document
2. The uploaded document was supplied together with the prompt instruction during generation
3. The LLM generated patient- and family-oriented explanations by paraphrasing the guideline content in plain language

No embedding-based retrieval, vector database indexing, or automated selection of guideline passages was performed. Parameter tuning and advanced retrieval pipelines were not intentionally implemented to reflect a pragmatic clinical scenario in which health care professionals use commercially available LLMs with minimal technical customization [16].

### Selection of Generated Texts

For human evaluation, we compared the relative quality of practically usable outputs across model-prompt configurations. Because human evaluation requires substantial time and effort, the number of texts was restricted to a feasible sample size, and clearly incomplete outputs were excluded prior to evaluation.

**Figure 1.**
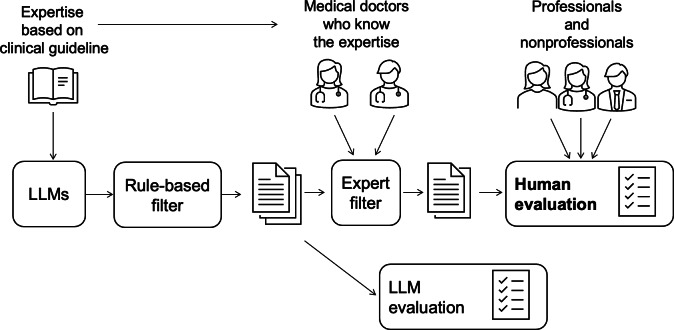
Flowchart of the selection process for the 9 final texts. A total of 72 texts were generated using 4 prompt designs across the 6 LLMs, with 3 outputs each. After excluding texts less than 500 characters (n=16) and those lacking explicit mention of PICS (n=3), 53 texts remained. The longest text was selected from each unique prompt-model pair (n=16). Following an expert review by 2 medical specialists, 7 texts were excluded, resulting in 9 final texts for analysis. LLM: large language model; PICS: post–intensive care syndrome.

The generated texts were selected through a structured 3-step process. First, 72 texts (6 models × 4 prompts × 3 outputs, Multimedia Appendix 2) were screened for minimum length (≥500 Japanese characters) and explicit mention of PICS, leaving 53 texts. Second, for each model-prompt combination, the longest output was selected as a proxy for informational coverage to reduce the likelihood of including incomplete or truncated responses and to ensure sufficient content for human evaluation, resulting in 21 texts. Third, to ensure balanced representation across prompts and models, four models (GPT-4, Llama3:7b, MedLLaMA, and Mistral) were retained. Because all MedLLaMA outputs for prompt 3 had been excluded during initial screening, the longest excluded output was reinstated to maintain balance. This process yielded 16 final texts (4 models × 4 prompts, Multimedia Appendix 3). These texts were initially generated in English and subsequently formatted into Japanese A4-sized informational leaflets using a GPT-4o–based system. The translation quality of GPT-4 has been evaluated in prior research against human translators and was found to be comparable to junior professional translators in terms of overall error rates, supporting the reliability of this approach [17]. Subsequently, 2 board-certified emergency physicians (NH and TO) independently reviewed the 16 leaflets for accuracy, clarity, and completeness. Any disagreements between the reviewers were resolved through discussion to reach a consensus. Nine leaflets were selected for further evaluation (Figure 1, Table 1, and Multimedia Appendix 4). A few were excluded because of limited informational content or language that was more suited for medical professionals; however, there were no critical errors that could potentially cause harm to patients or their families. Details of the screening process and the rationale for expert-based selection are provided in Multimedia Appendix 5.

Ten individuals were recruited as evaluators: 2 nonmedical individuals, 2 health care providers (1 nurse and 1 physical therapist), 4 specialists with expertise in PICS (3 emergency physicians and 1 physical therapist), and 2 nonspecialist physicians without PICS-specific expertise. These evaluators were requested to assess 9 selected texts in terms of clarity, readability, and other factors, using 10 evaluation criteria. The evaluation framework was developed with reference to prior large-scale LLM assessment studies in medicine, including the approach described by Singhal et al [4].

**Table 1.** Overview of the 9 prompts (word count, characters, syllables, sentences).

Serial number	Prompt	Large language model	Number of outputs
1	Prompt 1 (zero-shot without the uploaded context)	Chat GPT-4o	1
2	Prompt 2 (zero-shot with the uploaded context)	Chat GPT-4o	1
3	Prompt 4 (step-by-step with the uploaded context)	Chat GPT-4o	1
4	Prompt 1 (zero-shot without the uploaded context)	LLaMA 3 70B	1
5	Prompt 3 (few-shot with the uploaded context)	LLaMA 3 70B	3
6	Prompt 4 (step-by-step with the uploaded context)	LLaMA 3 70B	1
7	Prompt 1 (zero-shot without the uploaded context)	MedLLaMA2	3
8	Prompt 1 (zero-shot without the uploaded context)	Mistral	1
9	Prompt 4 (step-by-step with the uploaded context)	Mistral	3

The survey was conducted using Google Forms and included the following components.

1. Evaluator demographics: age, sex, profession, and area of expertise
2. Evaluation items rated on a 10-point Likert scale:
  - Scientific or clinical validity: how scientifically and clinically appropriate is the text?
  - Degree of harmfulness: to what extent could the content be harmful?
  - Frequency of harmfulness: how likely is it that harm could result from this content?
  - Readability: how comprehensible is the text?
  - Accuracy: is the text medically accurate?
  - Logical consistency: is the text logically structured?
  - Difficulty to read: how difficult is the text to read?
  - Inappropriateness: does the text include inappropriate content?
  - Omissions: are any essential elements missing from the text?
  - Potential for bias: does the content include information that may not be applicable to medical populations?

Because higher raw scores in degree of harmfulness, frequency of harmfulness, inappropriateness, omissions, and potential for bias represented greater risk or lower quality, these items were reverse-scored prior to analysis (transformed as 11—original score) so that higher scores consistently indicated better performance across all domains.

### LLM-Based Evaluation

In addition to human evaluation, LLM-based assessments were conducted using GPT-4o for structured comparison with human ratings. Two role-based prompts were used: “You are a medical doctor with extensive experience in emergency care” (physician-role) and “You are a family member of a patient in the intensive care unit” (patient-role).

The 9 selected texts were assessed using the following 6 aggregated dimensions:

- Agreement with scientific consensus
- Possibility and likelihood of harm
- Evidence of comprehension
- Reasoning and retrieval ability
- Presence of inappropriate, incorrect, or missing content
- Possibility of bias in the response

Among these, harm-related dimensions, presence of inappropriate or incorrect or missing content, and possibility of bias were reverse-scored (transformed as 11—original score) so that higher values consistently indicated better performance.

### Statistical Analysis

Objective assessments of the LLM-generated texts were conducted using human evaluations across 10 predefined criteria and AI-based evaluations incorporating 6 components. Subgroup analyses were performed to compare the evaluation metrics according to the prompt type, LLM used, and the use of text-augmented prompting approach. For graphical presentation of the evaluation results, 95% CIs were estimated using cluster bootstrapping with resampling at the evaluator level (5000 iterations). Additionally, we examined the differences in the evaluation scores according to age, sex, profession, and area of expertise.

As an exploratory analysis, all 72 generated texts (6 models × 4 prompts × 3 outputs) were additionally evaluated using the same GPT-4o–based assessment framework. This analysis aimed to examine evaluation patterns across multiple outputs within each model-prompt pair. To account for clustering of outputs within model-prompt pairs, a mixed-effects model was applied with model-prompt combinations specified as random intercepts. In addition, the mean scores of the 3 outputs within each model-prompt pair were calculated and analyzed using the same framework. Given the limited concordance observed between human and LLM-based evaluations, these analyses were considered exploratory and descriptive.

To assess the interevaluator variability between the human and LLM evaluators, we calculated the weighted κ across the 3 groups (human evaluators, GPT-generated responses simulating a medical doctor, and GPT-generated responses simulating a family member). Interrater reliability among the 10 human evaluators was assessed using an intraclass correlation coefficient (ICC). A 2-way mixed-effects model with absolute agreement (ICC [A,10]) was applied to evaluate consistency across the 10 evaluation criteria.

Categorical variables are reported as absolute numbers and percentages, whereas continuous variables are expressed as mean (SD) or median (interquartile range), as appropriate. All statistical analyses were performed using GraphPad Prism 10 (GraphPad Software), pandas (v1.0.5), numpy (v1.21.4), seaborn (v0.11.2), and matplotlib (v3.5.1) tools in Python (v3.9.0).

### Ethical Considerations

This study involved human evaluators who assessed text materials generated by LLMs; however, the evaluators were not the research participants of the investigation. The study focused on the evaluation of AI-generated explanatory texts rather than on human participants themselves. No personal or identifiable information was collected from the evaluators. According to the *Ethical Guidelines for Medical and Health Research Involving Human Subjects* issued by the Ministry of Education, Culture, Sports, Science and Technology; the Ministry of Health, Labour and Welfare; and the Ministry of Economy, Trade and Industry of Japan (2017, amended 2021) [18], this type of research is considered outside the scope of studies requiring institutional review board approval because it does not involve interventions, collection of personal data, or research targeting human participants.

Therefore, institutional review board approval and formal informed consent were not required.

## Results

### Characteristics of Human Evaluators

Ten individuals evaluated the generated texts. Among them, 5 (50%) specialized in emergency and critical care medicine, 1 (10%) in internal medicine, 1 (10%) in psychiatry, and 3 (30%) in other fields. Two (20%) evaluators were involved in the development of the clinical practice guidelines for PICS (Table 2).

**Table 2.** Characteristics of human evaluators (N=10).

Characteristics	Human evaluators, n (%)
Sex	
Male	6 (60)
Female	4 (40)
Age (y)	
30‐34	4 (40)
35‐39	3 (30)
40‐44	2 (20)
45‐49	1 (10)
Occupation	
Physician	5 (50)
Nurse	1 (10)
Physical therapist	2 (20)
Nonmedical personnel	2 (20)
Specialty	
Emergency and critical care medicine	5 (50)
Internal medicine	1 (10)
Psychiatry	1 (10)
Others	3 (30)
Involvement in the guideline development	
Yes	2 (20)
No	8 (80)

### Human Evaluations

**Figure 2.**
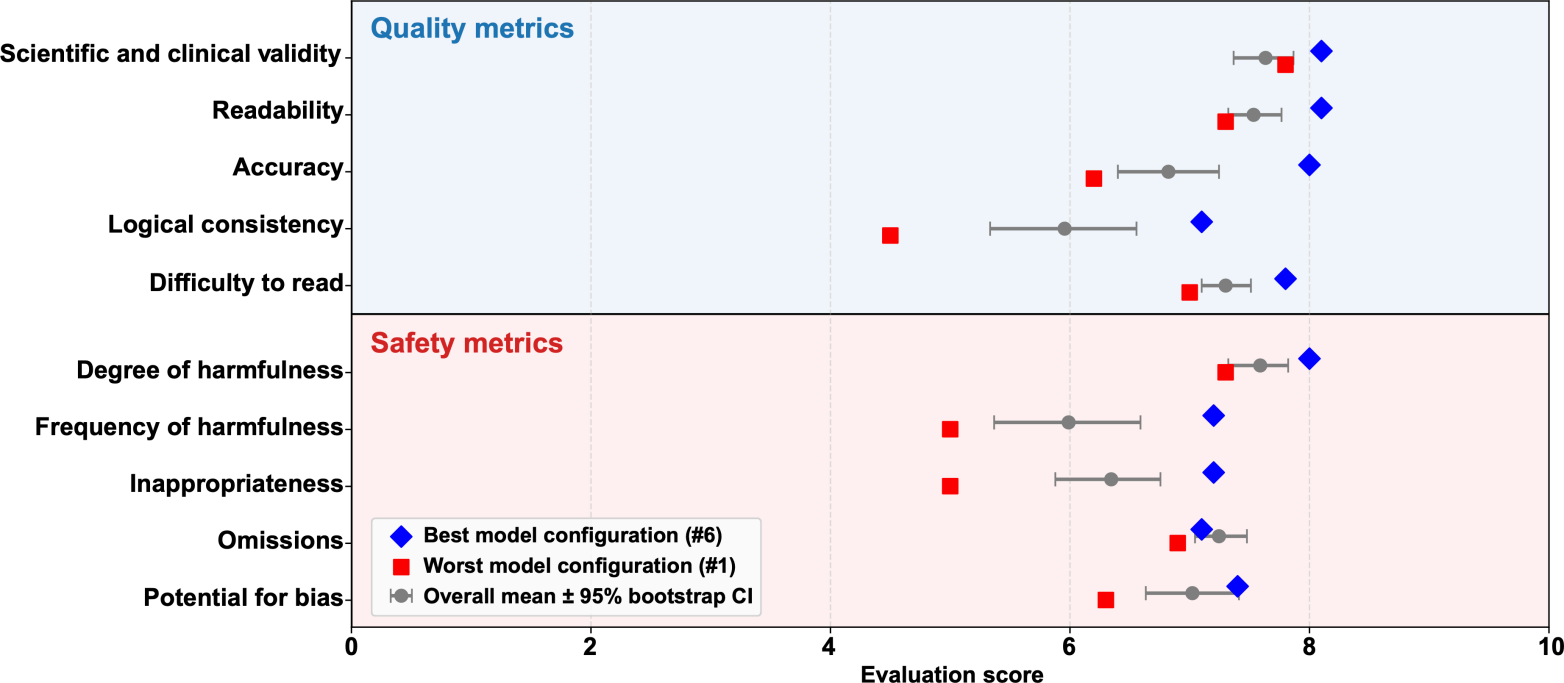
Human evaluation scores for the 9 selected texts across 10 evaluation criteria. Ten human evaluators assessed the 9 selected explanatory texts using 10 predefined evaluation criteria covering both informational quality and safety-related aspects. The forest plot shows the overall mean score for each criterion across the evaluated texts, with error bars representing 95% bootstrap CIs. Quality-related items are displayed in the upper panel, while safety-related items are shown in the lower panel to improve interpretability. Blue diamonds indicate the model configuration with the highest mean score among the evaluated texts, whereas red squares indicate the configuration with the lowest mean score. For negatively framed items (ie, degree of harmfulness, frequency of harmfulness, inappropriateness, omissions, and potential for bias), scores were reversed so that higher values consistently indicated better performance across all evaluation dimensions.

All selected texts received mean scores of 6.8 or higher across all evaluation items. Among the 9 outputs, the text generated by LLaMA 3 70B using a step-by-step approach combined with the uploaded context (prompt 4) had the highest overall mean score in the human evaluations. In contrast, the text produced using a simple zero-shot prompt and without the uploaded context (prompt 1) had the lowest overall mean score. These comparisons are descriptive and should be interpreted with caution given the limited number of texts per prompt condition. Items related to readability, difficulty in reading, and omission tended to receive comparatively lower ratings than the other evaluation domains (Figure 2).

The interrater reliability among the 10 human evaluators was high. The ICC indicated strong agreement (ICC[A,10]=0.903, 95% CI 0.869‐0.930; *P*<.001), indicating strong consistency in the application of the 10 evaluation criteria across evaluators.

### Assessments Using an LLM

GPT-4 evaluations indicated that the text generated by ChatGPT-4o using a step-by-step approach combined with the uploaded context (prompt 4) had comparatively higher scores for readability and scientific soundness among the evaluated outputs, which was broadly consistent with patterns observed in the human evaluations. By contrast, prompt 1 (zero-shot without the uploaded context) tended to receive lower ratings (Multimedia Appendix 6). The personalized GPT evaluations simulating a medical doctor and a family member showed substantial agreement (weighted κ coefficient: +0.818).

### Subgroup Analyses of LLMs and Prompt Engineering Strategies by Human Evaluators

**Figure 3.**
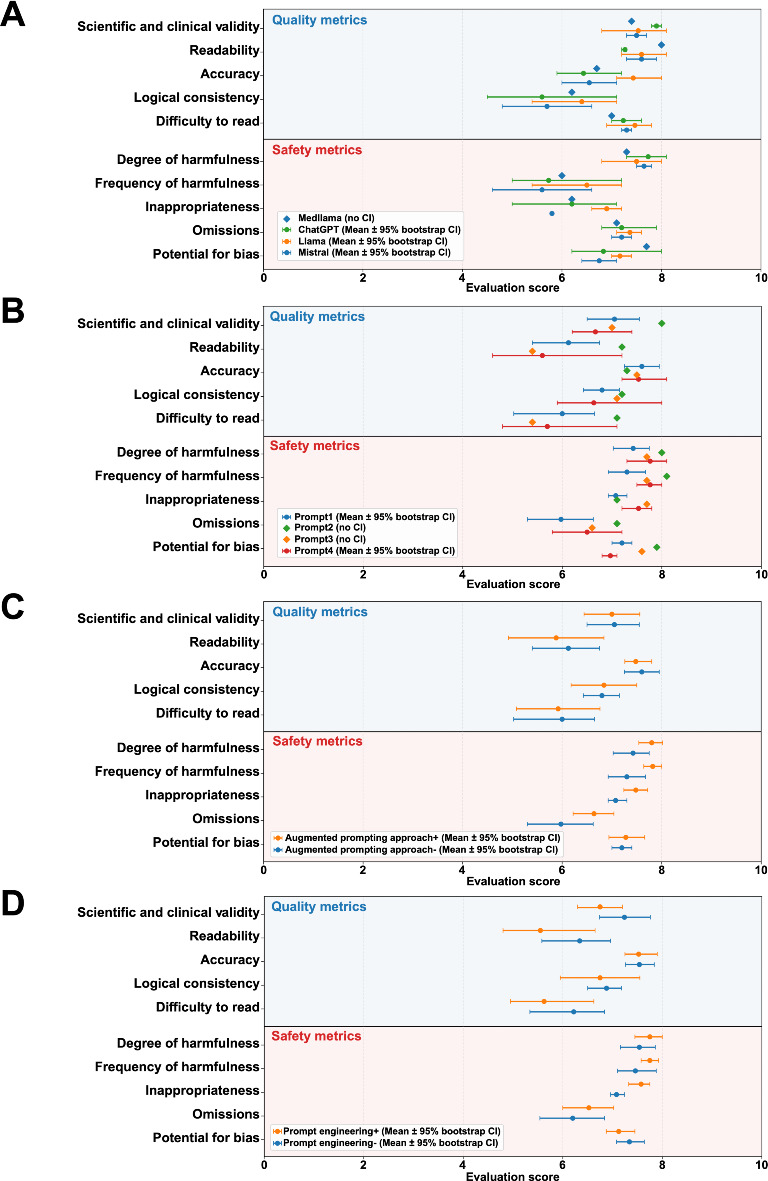
Subgroup analyses of human evaluation scores according to model and prompting strategies. Evaluations by 10 human evaluators for the 9 selected explanatory texts are presented according to (A) large language models, (B) prompt design, (C) the use of a text-augmented prompting approach based on clinical guidelines, and (D) prompt engineering strategies. The forest plots show the mean scores for each evaluation item across subgroups, with error bars representing 95% bootstrap CIs. Evaluation items related to informational quality are presented in the upper panels, whereas safety-related items are shown in the lower panels to facilitate visual distinction. For negatively framed items (ie, degree of harmfulness, frequency of harmfulness, inappropriateness, omissions, and potential for bias), scores were reversed so that higher values consistently indicated better performance across all evaluation dimensions.

For the LLM models, outputs generated using LLaMA 3 70B and Mistral tended to receive comparatively higher scores in domains such as scientific or clinical validity, accuracy, and readability. Among the prompt categories, prompt 2 (zero-shot with the uploaded context) appeared to show comparatively higher scores across multiple dimensions, whereas prompt 1 (zero-shot without the uploaded context) showed lower scores. Outputs generated using the uploaded context tended to receive higher scores for scientific validity and lower scores for omissions. Similarly, the use of prompt engineering strategies tended to be associated with higher scores for accuracy and readability and lower difficulty-in-reading ratings (Figure 3).

### Subgroup Analyses of Human Evaluation According to Evaluator Characteristics

Although some variability was observed across age groups, female evaluators tended to assign slightly higher scores than male evaluators. Physicians tended to provide marginally lower overall scores than nonmedical evaluators. No clear differences in evaluation scores were observed according to direct involvement in the development of the PICS guidelines (Multimedia Appendix 7).

### Interrater Variability Between Human and LLM Evaluators

**Figure 4.**
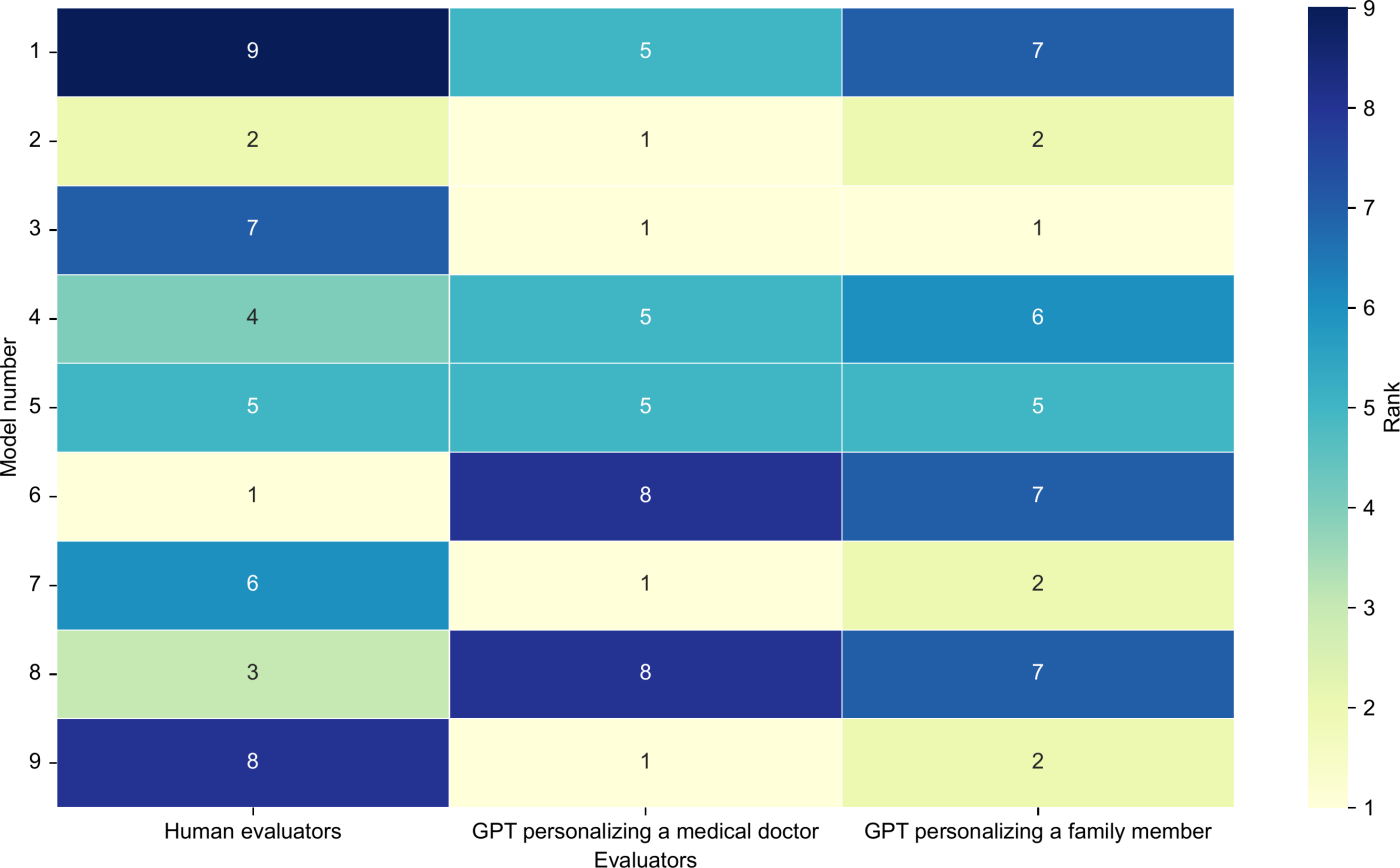
Interevaluator variability between human and artificial intelligence evaluators. This figure illustrates the interrater variability in total evaluation scores between human and artificial intelligence evaluators (simulating a medical doctor and a family member). Each row represents a different model (1-9), and each column represents the rank assigned by a different type of evaluator: human evaluators, the GPT simulating a medical doctor, and the GPT simulating a family member. The ranking scores ranged from 1 (best) to 9 (worst). Darker colors indicate higher ranks (worse evaluations), whereas lighter colors indicate better performance. GPT: generative pretrained transformer.

Variability between human and AI evaluations was observed, particularly for the highest- and lowest-ranked texts. Weighted κ coefficients suggested low-to-negative agreement (human vs GPT simulating a medical doctor −0.423; human vs GPT simulating a family member −0.276; Figure 4). Nevertheless, texts that were ranked highly by both human and LLM evaluators tended to have been generated using a step-by-step approach combined with text-augmented prompting, although the specific LLM models differed.

### Mixed-Effects Model Analysis of LLM-Based Evaluations Across 72 Generated Texts

The 9 texts selected through the screening process were among the higher-scoring outputs based on the LLM-based evaluation of all 72 generated texts (MultimediaAppendices 8^10^). In mixed-effects models including all 72 texts (MultimediaAppendices 11  12), Gemma, MedLLaMA, and Meditron:7b showed significantly lower mean scores compared with LLaMA3 70B in both patient- and physician-simulated evaluations. ChatGPT-4o and Mistral did not show statistically significant differences. Prompt engineering strategies were not significantly associated with scores in these models. Sensitivity analyses using mean scores across the three repeated outputs demonstrated similar directional trends (MultimediaAppendices 13  14).

## Discussion

### Principal Findings

In this study, information leaflets on PICS generated by commercially available LLMs for patients and their families were acceptable in terms of feasibility and validity. Among the 9 selected texts, those created using prompt engineering and text-augmented prompting approach tended to receive comparatively higher scores for readability, scientific accuracy, and overall quality, as assessed by human and AI evaluators. Subgroup analyses suggested that nonmedical evaluators tended to assign higher scores. Notably, the evaluation results from GPT-4 did not align with the human ratings. These findings should be interpreted as descriptive, given the limited number of texts in each subgroup.

### Comparison With Prior Work

A key strength of this study is that assessments were performed by nonmedical individuals and multidisciplinary health care professionals, including physicians with expertise in relevant fields. Although the information leaflets were intended for patients and their families, validation by medical professionals is essential to ensure content accuracy and appropriateness. Another notable strength of this study was the use of commercially available LLMs with text-augmented prompting approach to generate these materials. Although these models typically lack domain-specific knowledge, constructing a specialized LLM requires substantial resources. Our findings suggest that commercially available LLMs can produce content of satisfactory quality, thereby potentially facilitating their integration into clinical settings.

This study highlights the critical influence of LLM selection on the quality of information leaflets related to PICS. Given the diversity of their training datasets, LLMs inherently produce variable output quality [19,20]. A previous study evaluated the performance of 4 LLMs in generating therapeutic recommendations across 3 medical specialties and revealed significant differences in quality, accuracy, and potential harm. Notably, GPT-4 demonstrated a high level of alignment with physician assessments and proved to be effective in automating these evaluations [21]. Another study reported that GPT-4 outperformed other LLMs and human responses in generating accurate, relevant, helpful, and safe answers to patient questions regarding laboratory test results [22]. In our study, the LLaMA 3 70B and Mistral models tended to produce comparatively higher-quality outputs, which may be an important factor influencing performance.

In addition, we explored the impact of prompt engineering using strategies, including zero-shot, few-shot, and step-by-step approaches. Prompt engineering strategies can significantly affect the accuracy, integrity, and overall quality of the generated content [23]. Our results demonstrated that texts generated using simplistic zero-shot prompts without the uploaded context tended to receive lower scores across all evaluation criteria. In contrast, the step-by-step approach, which was previously reported to improve the output quality [24], tended to show comparatively higher scores. Moreover, the integration of retrieval-augmented generation has been reported to enhance factual accuracy and reduce content omissions [10]. Integrating structured guideline reformatting and advanced prompt engineering strategies using the GPT-4 Turbo significantly improved the accuracy of clinical decision support in chronic hepatitis C management, with the guideline context and formatting demonstrating more effectiveness than few-shot learning [25]. Taken together, these findings suggest the potential importance of using structured prompts and the integration of external knowledge retrieval in optimizing LLM performance in health care communications.

Subgroup analyses revealed significant differences in the evaluation scores based on evaluator characteristics. Female evaluators typically assigned higher scores than their male counterparts, which may reflect potential gender-related differences in the perception of readability or clarity [26]. In addition, physicians consistently rated the texts lower than nonmedical evaluators, which may reflect stricter professional standards or a more critical appraisal of clinical accuracy and completeness [27]. No clear differences in evaluation scores were observed according to direct involvement in the development of the PICS guidelines. This may support the validity and objectivity of the proposed evaluation methodology. The documents generated using the LLM were more acceptable to nonmedical individuals, suggesting that these texts may be relatively easier to use for communication with patients and their families. However, owing to the variation in ratings among evaluators, it is important to consider that perceptions may differ depending on the medical experience of the patients or their families.

Interevaluator variability analyses highlighted key differences between human- and AI-based assessments. Although both human and AI evaluators tended to rank outputs generated using step-by-step prompting combined with text-augmented prompting approach more favorably, variability was observed in the assessment of texts at extremes of performance, particularly in terms of scientific validity, readability, and overall appropriateness. Notably, the use of prompts simulating a family member improved the agreement between the LLMs and human experts, although the degree of alignment varied across the subscales. This observation suggests that the complexity of the evaluation content and the quality of the generated outputs can influence agreement levels, emphasizing the importance of careful prompt engineering and caution when relying solely on AI-based assessments [28]. Similar trends have been observed in clinical evaluations. For instance, when comparing ChatGPT’s assessment of clinical cases with evaluations by 2 board-certified physicians, only moderate interevaluator reliability was achieved, with notable discrepancies in domains such as diagnosis and treatment decisions, highlighting the risk of overreliance on AI in sensitive medical contexts [29]. Future studies should further investigate the factors contributing to human-AI discrepancies and optimize methodologies to enhance the alignment between AI-generated evaluations and expert human judgments.

Effective communication is a key determinant of patient satisfaction. Patient satisfaction is strongly associated with the quality of physician-patient communication and that of discharge planning, thus emphasizing the importance of interpersonal engagement in clinical care [30]. In recent years, patients have increasingly turned to the internet and digital media to obtain health-related information independently [31]. Although such information-seeking behavior empowers patients, it introduces new communication challenges for clinicians, who must address misinformation and convey accurate explanations effectively under limited timeframes. Integrating commercially available LLMs into health care communication workflows may help bridge this gap by enabling patients to interact with reliable, understandable, and personalized explanations, potentially enhancing their engagement and satisfaction.

### Limitations

This study has several limitations.

First, all the generated texts focused exclusively on PICS, limiting the generalizability of the findings to other clinical subjects.

Second, a deliberate design choice in this study was to prioritize real-world feasibility over technical optimization. Accordingly, we used default generation parameters and a simple document-upload approach rather than implementing a fully indexed retrieval-augmented generation pipeline, reflecting how health care professionals are most likely to use commercially available LLMs in routine clinical practice. Furthermore, the use of a default 2048-token context window represents a technical limitation. We did not verify whether the full text of the uploaded guidelines was retained in the context window during generation. Consequently, partial truncation may have occurred, potentially attenuating the benefits of context-augmented prompting. While this limits the internal validity of the models’ theoretical maximum performance, it accurately reflects real-world deployment conditions, where clinicians typically rely on default system settings without control over context management. In addition, the instruction to “search” in prompt 4 was semantically ambiguous given the offline environment. While our functional intent was to enforce reliance on the uploaded context rather than pretrained knowledge, the explicit use of the term “search”—instead of “extract” or “summarize”—may have inadvertently triggered retrieval-simulation behaviors or role-playing. Since the generated texts were evaluated based on this prompt, we cannot rule out the possibility that this phrasing influenced the stylistic presentation or narrative structure of the outputs. This discrepancy represents a methodological limitation regarding prompt design.

Third, no formal sample size calculation was performed because this was a pilot study intended to explore preliminary trends. The human evaluation represents a comparative assessment among outputs, meeting a minimal threshold of completeness rather than the full distribution of all generated texts. The selection process was not statistically driven but was designed to ensure the feasibility of human evaluation. In addition, the selection procedures, including the use of the longest output as a proxy for informational coverage and the expert-based filtering prior to evaluation, may have preferentially retained more complete or higher-quality texts while excluding less suitable outputs. As a result, the evaluated sample may not fully represent the distribution of all generated texts and may have led to an overestimation of overall quality. Therefore, the findings should not be interpreted as estimates of overall model performance, but rather as exploratory comparisons across model-prompt configurations under feasibility-constrained conditions. Moreover, the final human evaluation was based on 9 selected texts. Certain prompts (prompts 2 and 3) were represented by only a single text (n=1). Such small and uneven group sizes substantially limit statistical power and the reliability of detecting meaningful differences across prompt strategies. Therefore, these comparisons are exploratory and descriptive and do not allow definitive conclusions regarding the superiority of any specific prompt configuration.

Fourth, the number of human evaluators was relatively small, which may have affected the representativeness of the findings. Although interevaluator reliability was formally assessed using an ICC, residual variability across evaluators and evaluation items may still have remained. Furthermore, discrepancies were observed between human and LLM-based evaluations; however, the limited sample size made it difficult to systematically examine the structural factors underlying these differences. Future studies using larger datasets are needed to investigate the mechanisms driving divergence between human and LLM assessments and to develop AI-based evaluation methods that more accurately reflect human judgment patterns.

Fifth, LLM outputs were generated in English and subsequently translated into Japanese using a GPT-4–based system prior to evaluation. Accordingly, human evaluators assessed the translated Japanese texts rather than the original English outputs. Although translations were performed without formal human postediting, prior studies suggest that GPT-4 achieves translation quality comparable to that of junior professional translators [17], supporting the general adequacy of this approach. However, subtle linguistic or cultural nuances may not have been fully preserved. In addition, the GPT-4–based translation process may have systematically normalized sentence structure and wording, potentially increasing readability- and clarity-related scores, independent of the intrinsic quality of the source LLM outputs. Therefore, evaluation scores—particularly for domains such as readability, clarity, and difficulty of reading—may reflect not only the quality of the LLM-generated content but also the characteristics of the translation process, which may complicate the attribution of differences to individual models or prompt strategies. In this context, at the time of the study, several commercially available LLMs exhibited more stable performance in English than in Japanese, which motivated the decision to generate outputs in English prior to translation. While this approach reflects a pragmatic real-world workflow, it introduces an additional processing layer that may affect the interpretability. With recent advances in multilingual capabilities and agent-based control of LLMs, direct generation in Japanese has become increasingly feasible. Future studies should therefore consider evaluating outputs generated natively in the target language to better isolate the effects of model architecture and prompt design. In addition, because safety-related domains consistently received high scores, their inclusion in the composite score may have obscured differences in quality-related scores. To enhance transparency, safety and quality domains were additionally presented separately in the figure.

Finally, patients and their family members were not included in the evaluation process, which may limit the real-world applicability of our findings. For clinical translation, incorporating direct feedback from a target audience would provide stronger evidence of clinical effectiveness.

### Conclusions

Among the selected texts included in the final human evaluation, informational materials generated using commercially available LLMs were generally rated as acceptable by human evaluators, and none contained harmful content. These findings suggest that LLMs may support the development of patient-facing informational materials under feasibility-constrained conditions, although further validation with larger and more diverse samples is warranted.

## Supplementary material

10.2196/81606 Multimedia Appendix 1 Prompt design.

10.2196/81606 Multimedia Appendix 2 Generated content of 72 texts.

10.2196/81606 Multimedia Appendix 3 Generated content of 16 texts.

10.2196/81606 Multimedia Appendix 4 Generated content of 9 texts.

10.2196/81606 Multimedia Appendix 5 Detailed screening and expert selection process.

10.2196/81606 Multimedia Appendix 6 Role-based comparison of large language model (LLM) evaluation scores for the 9 selected texts. Evaluation scores generated through LLM-based assessment were compared according to simulated evaluator roles: (A) a family member of an intensive care unit patient and (B) a medical doctor. The forest plots show the mean scores for each evaluation item across the 9 selected texts, with error bars representing 95% bootstrap CIs. Evaluation items related to informational quality are presented in the upper panels, whereas safety-related items are shown in the lower panels to facilitate visual distinction. Blue diamonds indicate the highest-scoring configuration among the evaluated texts, and red squares indicate the lowest-scoring configuration.

10.2196/81606 Multimedia Appendix 7 Subgroup analyses of total evaluation scores according to evaluator characteristics.

10.2196/81606 Multimedia Appendix 8 Comparison of evaluation scores between all 72 generated texts and the final 9 selected texts. Evaluation scores generated through large language model–based assessment were compared between all 72 generated texts and the final subset of 9 selected texts, based on evaluations performed from the perspectives of (A) a simulated family member of an intensive care unit patient and (B) a simulated medical doctor. The forest plots show the mean scores for each evaluation item, with error bars representing 95% bootstrap CIs. Evaluation items related to informational quality are presented in the upper panels, whereas safety-related items are shown in the lower panels to facilitate visual distinction. Blue markers indicate the mean scores of the selected subset of 9 texts, whereas orange markers indicate the overall mean scores across all 72 generated texts.

10.2196/81606 Multimedia Appendix 9 Large language model (LLM)–based patient-role evaluation results for all 72 generated texts. This table presents the evaluation results of all 72 generated texts assessed by an LLM simulating a patient role across 6 predefined criteria. The texts are ranked in descending order according to their total scores. The final subset of 9 selected texts is highlighted in orange, and their corresponding ranking positions among all 72 texts are indicated.

10.2196/81606 Multimedia Appendix 10 Large language model (LLM)–based physician-role evaluation results for all 72 generated texts. This table presents the evaluation results of all 72 generated texts assessed by an LLM simulating a physician role across 6 predefined criteria. The texts are ranked in descending order according to their total scores. The final subset of 9 selected texts is highlighted in orange, and their corresponding ranking positions among all 72 texts are indicated.

10.2196/81606 Multimedia Appendix 11 Mixed-effects model analysis of large language model–based patient-role evaluations across all 72 texts.

10.2196/81606 Multimedia Appendix 12 Mixed-effects model analysis of large language model–based physician-role evaluations across all 72 texts.

10.2196/81606 Multimedia Appendix 13 Sensitivity analyses using large language model–based patient-role evaluations of all 72 texts (mean of 3 outputs).

10.2196/81606 Multimedia Appendix 14 Sensitivity analyses using large language model–based physician-role evaluations of all 72 texts (mean of 3 outputs).

## Abbreviations

AI: artificial intelligence
ICC: intraclass correlation coefficient
LLM: large language model
PICS: post–intensive care syndrome
