# Supplementary material for: Use of Commercially Available Large Language Models to Generate Information Leaflets on Post–Intensive Care Syndrome: Clinical Utility Assessment
Source: JMIR Form Res. 2026 May 14;10:e81606. doi: 10.2196/81606 (PMC13175452; doi:10.2196/81606)
Supplement: Multimedia Appendix 1 [file formative-v10-e81606-s001.docx]

**Prompt1**

Your role is to convey medical guidelines in an easily understandable manner to various professionals. This time, the target audience is patients and their families. Please explain PICS (Post-Intensive Care Syndrome).

**Prompt2**

You are tasked with explaining medical guidelines to various professionals in an easy-to-understand manner. This time, the target audience is patients and their families. Please paraphrase the attached guidelines to explain PICS (Post-Intensive Care Syndrome).

**Prompt3**

"You are responsible for explaining PICS (Post-Intensive Care Syndrome) to patients and their families based on medical guidelines. Below are some specific questions and exemplary answers. Please use these as a reference to provide clear and understandable answers to patients and their families.

Question 1: Who is more likely to develop PICS?

Exemplary Answer 1: PICS is more likely to occur in patients who have been admitted to the ICU due to severe illnesses or injuries. Conditions like sepsis, severe respiratory failure, and shock often lead to ICU admissions, subsequently increasing the risk of developing PICS. Elderly patients are particularly prone to developing PICS.

Question 2: How can PICS be prevented?

Exemplary Answer 2: Early rehabilitation and proper mental support are crucial in preventing PICS. Starting physical rehabilitation early during the ICU stay and ensuring that both patients and their families receive psychological support can be effective. Continuous follow-up after ICU discharge is also recommended. It is important to maintain close communication with the medical team and provide ongoing information through leaflets and other resources.

Question 3: How can families support a loved one with PICS?

Exemplary Answer 3: Families can provide essential emotional support to patients experiencing PICS. Listening to the patient when they feel anxious or depressed and helping them access professional counseling if needed can be beneficial. Additionally, families should be aware of PICS-F (family PICS) and take care of their own mental health as well.

Question 4: What is PICS?

Answer:"

**Prompt4**

""You are responsible for explaining medical guidelines to various professionals in an easy-to-understand manner. This time, the target audience is patients and their families. Based on the attached guidelines, please explain PICS (Post-Intensive Care Syndrome) in a clear and understandable way. Additionally, provide detailed information on the causes, symptoms, and treatment methods of PICS, along with specific examples and advice for daily life that patients and their families can easily understand. Please follow the phases below:

Search Phase: Search for the latest clinical guidelines and reliable sources of information on PICS to gather detailed information on its causes, symptoms, and treatment methods. Also, collect information on specific examples and advice for daily life that are easy for patients and families to understand.

Generation Phase: Based on the information obtained from the search phase, create a clear and understandable explanation of PICS. Include detailed information on the causes, symptoms, and treatment methods, along with practical advice that will be useful for patients and their families.""
