# Supplementary material for: Use of Commercially Available Large Language Models to Generate Information Leaflets on Post–Intensive Care Syndrome: Clinical Utility Assessment
Source: JMIR Form Res. 2026 May 14;10:e81606. doi: 10.2196/81606 (PMC13175452; doi:10.2196/81606)
Supplement: Multimedia Appendix 4 [file formative-v10-e81606-s004.docx]

**Text 1**

**ChatGPT4o_Prompt1_1**

Sure! Here is a simple explanation of Post-Intensive Care Syndrome (PICS) for patients and their families:
---
**Understanding Post-Intensive Care Syndrome (PICS)**

**What is PICS?**
Post-Intensive Care Syndrome, or PICS, is a collection of health problems that some people experience after they have been treated in an intensive care unit (ICU). It can affect both the mind and the body.

**Who is at risk?**
Anyone who has spent time in an ICU can develop PICS, but it is more common in people who have been in the ICU for a long time, had severe illnesses, or needed a lot of medical support, such as breathing machines.

**What are the symptoms?**
PICS can cause a range of symptoms that fall into three main categories:

1. **Physical Symptoms:**
 - Muscle weakness and fatigue
 - Problems with balance and coordination
 - Shortness of breath

2. **Mental Health Symptoms:**
 - Anxiety and depression
 - Post-traumatic stress disorder (PTSD)
 - Sleep problems

3. **Cognitive Symptoms:**
 - Memory problems
 - Difficulty concentrating
 - Trouble with planning and organizing

**Why does PICS happen?**
PICS occurs because the body and brain undergo significant stress during severe illness and intensive treatment. The treatments that save lives in the ICU can sometimes have side effects that lead to these long-term issues.

**How is PICS treated?**
Treatment for PICS involves a combination of medical care, physical therapy, mental health support, and cognitive rehabilitation. Here is how each part helps:

- **Medical Care:** Regular check-ups with your doctor to monitor and manage physical symptoms.
- **Physical Therapy:** Exercises to strengthen muscles, improve balance, and increase endurance.
- **Mental Health Support:** Counseling or therapy to help with anxiety, depression, and PTSD.
- **Cognitive Rehabilitation:** Activities and strategies to improve memory and thinking skills.

**What can patients and families do?**
- **Stay Informed:** Learn about PICS and what to expect during recovery.
- **Seek Support:** Don’t hesitate to ask for help from healthcare professionals, support groups, and family members.
- **Be Patient:** Recovery from PICS can take time. Celebrate small improvements and stay positive.

**Remember:** PICS is a recognized condition, and there is support available. With the right care and support, many people recover and regain their quality of life.
---
If you have any specific questions or need further details, feel free to ask!

**Text 2**

**ChatGPT4o_Prompt2_1**

**Understanding Post-Intensive Care Syndrome (PICS)**

**What is PICS?**
Post-Intensive Care Syndrome (PICS) includes physical, mental, and cognitive problems that occur during or after a stay in an intensive care unit (ICU). These issues can affect not only the patient but also their families.

**Why is PICS Important?**
Advancements in ICU care have improved survival rates, but many patients experience long-term effects after ICU discharge. Studies show that a significant number of patients face challenges in physical, mental, and cognitive health up to a year after leaving the ICU.

**Symptoms of PICS:**
1. **Physical Symptoms:** Weakness, fatigue, difficulty moving.
2. **Mental Health Symptoms:** Anxiety, depression, PTSD (post-traumatic stress disorder).
3. **Cognitive Symptoms:** Memory problems, difficulty concentrating.

**Impact on Families:**
Families of ICU patients can also experience mental health issues, known as PICS-F (Post-Intensive Care Syndrome-Family). This can include anxiety, depression, and PTSD, often linked to the stress of having a loved one in intensive care.

**Preventing and Managing PICS:**
- **Early Rehabilitation:** Encouraged to start physical therapy early to prevent or reduce physical impairments.
- **Mental Health Support:** Providing psychological support and counseling to address anxiety, depression, and PTSD.
- **Cognitive Rehabilitation:** Activities and strategies to improve memory and thinking skills.

**How to Support Patients and Families:**
1. **Provide Information:** Healthcare providers should inform patients and families about PICS and PICS-F, both at ICU admission and discharge. This includes using leaflets and continuous communication.
2. **Follow-Up Care:** Post-ICU follow-up clinics can help monitor and support patients' physical, mental, and cognitive recovery.
3. **Home Visits:** Visits from ICU staff to the patient's home can help address ongoing health issues and provide support.

**Effective Communication:**
- **Two-Way Communication:** Ensure that patients and families can ask questions and express concerns.
- **Continuous Information:** Regular updates and follow-up can help patients and families feel informed and supported.

**Conclusion:**
PICS is a complex condition that requires a comprehensive approach to manage and prevent. With proper support and information, patients and families can better navigate the challenges following an ICU stay. Healthcare providers play a crucial role in ensuring that both patients and their families receive the care and information they need for a successful recovery.
---
If you have any specific questions or need further details, feel free to ask!

**Text 3**

**ChatGPT4o_Prompt4_1**

### Post-Intensive Care Syndrome (PICS): A Guide for Patients and Their Families

**What is PICS?**
Post-Intensive Care Syndrome (PICS) refers to a range of health problems that can occur after a patient has been treated in an intensive care unit (ICU). These problems can affect the patient's body, mind, and cognitive abilities, and can also impact the mental health of their families.

**Causes of PICS:**
PICS develops due to the severe stress and intensive treatment that patients undergo in the ICU. Common causes include:
- **Severe Illnesses:** Conditions like sepsis, severe respiratory failure, and shock.
- **Intensive Treatments:** Long periods on ventilators or other life-supporting devices.
- **Age and Pre-existing Conditions:** Elderly patients and those with pre-existing health conditions are more vulnerable.

**Symptoms of PICS:**
PICS symptoms are diverse and can be categorized into three main areas:

1. **Physical Symptoms:**
 - **Muscle Weakness:** Difficulty moving or performing everyday tasks.
 - **Fatigue:** Persistent tiredness and low energy levels.
 - **Breathing Problems:** Shortness of breath during activities.

2. **Mental Health Symptoms:**
 - **Anxiety and Depression:** Feelings of worry, sadness, and hopelessness.
 - **PTSD:** Experiencing flashbacks, nightmares, or severe stress related to ICU experiences.
 - **Sleep Problems:** Difficulty falling or staying asleep.

3. **Cognitive Symptoms:**
 - **Memory Issues:** Trouble remembering recent events or tasks.
 - **Concentration Difficulties:** Struggling to focus or stay on task.
 - **Planning and Organizing:** Finding it hard to plan activities or manage daily routines.

**Preventing and Treating PICS:**
Effective prevention and treatment of PICS involve early intervention and comprehensive care:

- **Early Rehabilitation:**
 - **Physical Therapy:** Begin exercises early to strengthen muscles and improve mobility.
 - **Occupational Therapy:** Helps patients regain the ability to perform daily activities.

- **Mental Health Support:**
 - **Counseling:** Professional help for dealing with anxiety, depression, and PTSD.
 - **Support Groups:** Joining groups of other ICU survivors for mutual support.

- **Cognitive Rehabilitation:**
 - **Memory Exercises:** Activities and games to enhance memory and cognitive function.
 - **Structured Routines:** Keeping a regular schedule to help with concentration and organization.

**Support for Families (PICS-F):**
Families can also experience mental health issues due to the stress of a loved one's ICU stay. Here are some ways families can support both their loved ones and themselves:

- **Emotional Support:** Be patient and listen to your loved one. Encourage open conversations about their feelings.
- **Seek Professional Help:** If needed, get professional counseling for both the patient and family members.
- **Stay Informed:** Learn about PICS and its symptoms to better understand what your loved one is going through.
- **Self-Care:** Take care of your own mental and physical health. Join support groups for families of ICU patients.

**Practical Advice for Daily Life:**

1. **Physical Activity:** Engage in light exercises as recommended by a healthcare provider to improve strength and stamina.
2. **Healthy Diet:** Maintain a balanced diet to support overall health and recovery.
3. **Rest and Relaxation:** Ensure adequate rest and relaxation to aid in physical and mental recovery.
4. **Routine Follow-Ups:** Attend all scheduled follow-up appointments with healthcare providers.
5. **Use Resources:** Utilize leaflets, websites, and other resources provided by healthcare teams to stay informed about PICS and its management.

**Conclusion:**
PICS is a challenging condition, but with the right support and care, patients and their families can manage the symptoms and improve their quality of life. Early intervention, comprehensive rehabilitation, and continuous support are key to successful recovery.

If you have any specific questions or need further details, feel free to ask!

**Text 4**

**llama3:70b_Prompt1_1**

I'd be happy to help explain Post-Intensive Care Syndrome (PICS) in a way that's easy for patients and their families to understand.

**What is Post-Intensive Care Syndrome (PICS)?**

PICS is a set of physical, emotional, and cognitive challenges that some people experience after being in an Intensive Care Unit (ICU). The ICU is a special department in the hospital where critically ill patients receive close monitoring and life-sustaining treatments.

**Why does PICS happen?**

When someone is in the ICU, their body goes through a lot of stress. They may be on machines to help them breathe, eat, or support their heart rate. They may also experience pain, anxiety, and sleep disturbances. This intense physical and emotional stress can lead to changes in the body and brain that affect how they feel and function after leaving the ICU.

**What are the symptoms of PICS?**

Symptoms of PICS can vary from person to person, but common experiences include:

1. **Physical symptoms**: Muscle weakness, fatigue, difficulty sleeping, pain, or numbness/tingling in hands and feet.
2. **Cognitive symptoms**: Difficulty concentrating, memory problems, or confusion.
3. **Emotional symptoms**: Anxiety, depression, mood swings, or feelings of hopelessness.
4. **Psychological symptoms**: Post-traumatic stress disorder (PTSD), hallucinations, or nightmares.

**How long does PICS last?**

The duration of PICS can vary greatly from person to person. Some people may experience symptoms for a few weeks, while others may struggle with them for months or even years after leaving the ICU.

**What can be done to prevent or manage PICS?**

While it's not possible to completely prevent PICS, there are steps that healthcare professionals and patients can take to reduce its impact:

1. **Early mobility**: Getting out of bed and moving around as soon as possible while in the ICU.
2. **Pain management**: Managing pain effectively to reduce discomfort and anxiety.
3. **Sleep promotion**: Creating a sleep-conducive environment and promoting relaxation techniques.
4. **Family support**: Involving family members in care and providing emotional support.
5. **Rehabilitation programs**: Participating in rehabilitation programs after leaving the ICU to improve physical function and address cognitive and emotional challenges.

**What can patients and families do?**

If you or a loved one has been in an ICU, it's essential to:

1. **Be aware of PICS**: Educate yourself about the potential risks and symptoms.
2. **Seek support**: Talk to healthcare professionals, family members, or support groups about your experiences and feelings.
3. **Prioritize self-care**: Focus on rest, nutrition, and stress management after leaving the ICU.
4. **Advocate for yourself or loved one**: Encourage healthcare providers to address any concerns or symptoms you may be experiencing.

Remember, PICS is a common experience for many people who have been in an ICU. With awareness, support, and proactive care, it's possible to manage its effects and improve overall well-being.

**Text 5**

**llama3:70b_Prompt3_3**

"Here's a comprehensive explanation of PICS (Post-Intensive Care Syndrome) that I can provide to patients and their families based on medical guidelines:
 
What is PICS?
 
PICS, or Post-Intensive Care Syndrome, refers to a set of physical, emotional, and cognitive symptoms that can occur in patients after they have been discharged from the Intensive Care Unit (ICU). These symptoms can affect not only the patient but also their family members.
 
Who is more likely to develop PICS?
 
PICS is more likely to occur in patients who have been admitted to the ICU due to severe illnesses or injuries. Conditions like sepsis, severe respiratory failure, and shock often lead to ICU admissions, subsequently increasing the risk of developing PICS. Elderly patients are particularly prone to developing PICS.
 
How can PICS be prevented?
 
Early rehabilitation and proper mental support are crucial in preventing PICS. Starting physical rehabilitation early during the ICU stay and ensuring that both patients and their families receive psychological support can be effective. Continuous follow-up after ICU discharge is also recommended. It is important to maintain close communication with the medical team and provide ongoing information through leaflets and other resources.
 
How can families support a loved one with PICS?
 
Families can provide essential emotional support to patients experiencing PICS. Listening to the patient when they feel anxious or depressed and helping them access professional counseling if needed can be beneficial. Additionally, families should be aware of PICS-F (family PICS) and take care of their own mental health as well.
 
What are the symptoms of PICS?
 
Symptoms of PICS may include:
 
Muscle weakness and fatigue
 
Cognitive impairment, such as memory loss or difficulty concentrating
 
Emotional distress, including anxiety, depression, and post-traumatic stress disorder (PTSD)
 
Sleep disturbances
 
Chronic pain
 
Difficulty with daily activities
 
 
How is PICS treated?
 
Treatment for PICS typically involves a multidisciplinary approach, including medical care, rehabilitation therapy, and psychological support. Medications may be prescribed to manage symptoms such as depression or anxiety. Rehabilitation therapies, such as physical, occupational, and speech therapy, can help improve functional abilities. Psychological counseling and support groups can also provide emotional support and coping strategies.
 
Remember, it's essential for patients and their families to work closely with the medical team to develop a personalized treatment plan that addresses their specific needs and symptoms.
"

**Text 6**

**llama3:70b_Prompt4_1**

"Here's an explanation of PICS (Post-Intensive Care Syndrome) in a clear and understandable way:

**What is PICS?**

PICS stands for Post-Intensive Care Syndrome. It refers to physical, cognitive, and mental disorders that can occur during or after Intensive Care Unit (ICU) stay, or even after discharge from the hospital. These disorders can affect not only patients who have been critically ill but also their families.

**Causes of PICS**

PICS can be caused by various factors, including:

* Sepsis, a severe infection that can cause organ failure
* ICU stay and treatment
* Age (older adults are more susceptible)
* Pre-existing medical conditions

**Symptoms of PICS**

The symptoms of PICS can vary from person to person, but common ones include:

* Physical symptoms: muscle weakness, fatigue, pain, and sleep disturbances
* Cognitive symptoms: memory loss, confusion, difficulty concentrating, and mood changes
* Mental health symptoms: anxiety, depression, post-traumatic stress disorder (PTSD), and fear

**Treatment Methods for PICS**

Treating PICS requires a comprehensive approach that involves healthcare providers, patients, and their families. Some effective treatment methods include:

* Medications to manage symptoms and prevent complications
* Rehabilitation programs to improve physical function and cognitive abilities
* Counseling and therapy to address mental health concerns
* Family support and education to promote caregiving and coping

**Practical Advice for Daily Life**

Here are some practical tips that patients and their families can follow to cope with PICS:

* Create a daily routine that includes rest, exercise, and social activities
* Prioritize self-care and seek emotional support from loved ones or support groups
* Break down tasks into smaller, manageable steps to reduce feelings of overwhelm
* Keep a journal or diary to track progress and identify patterns

**Example**

Let's consider an example. Mrs. Tanaka, a 70-year-old woman, was hospitalized for sepsis and spent two weeks in the ICU. After discharge, she experienced fatigue, memory loss, and anxiety. Her family noticed that she had difficulty performing daily tasks and was withdrawn from social activities. With the help of healthcare providers, Mrs. Tanaka started a rehabilitation program to improve her physical function and cognitive abilities. She also attended counseling sessions to address her anxiety and depression. Her family learned how to provide emotional support and assist with caregiving tasks. Through this comprehensive approach, Mrs. Tanaka was able to recover from PICS and regain her independence.

Remember, PICS is a common condition that can affect anyone who has been critically ill. By understanding its causes, symptoms, and treatment methods, patients and their families can take proactive steps to manage PICS and improve their quality of life."

**Text 7**

**Medllama_Prompt1_3**

"Dear Patients and Families,

We are here to help you better understand your health journey, including terms related to care after a critical illness or extended stay in an intensive care unit. Today, we will discuss Post-Intensive Care Syndrome (PICS).

Post-Intensive Care Syndrome is a term used for the group of physical, emotional, and cognitive problems that some people may experience following a critical illness or prolonged stay in the ICU. These complications can impact daily life, including mobility, mood, and thinking abilities.

Here's what you need to know about each type of PICS:

1. Physical PICS: This refers to ongoing physical problems such as weakness, fatigue, pain, or changes in appetite or sleep patterns. These symptoms may persist for weeks, months, or even years after leaving the ICU.

2. Psychological PICS: Survivors of critical illnesses may experience emotional challenges like anxiety, depression, post-traumatic stress disorder (PTSD), and adjustment disorders. If you notice changes in your emotional wellbeing after leaving the ICU, it is crucial to seek help from healthcare professionals.

3. Cognitive PICS: Some survivors may have difficulties with memory, concentration, or problem-solving abilities. This type of PICS is often referred to as ""brain fog"" and can significantly impact daily activities like work or managing finances.

PICS can affect anyone who has spent time in the ICU, regardless of age or severity of illness. It's essential to be aware of these potential complications so that you can take steps towards recovery and rehabilitation with your healthcare team.

Remember, recovering from a critical illness is about more than just getting better physically; it also involves addressing any psychological, cognitive, or emotional challenges that may arise. Open communication with your healthcare providers and loved ones is vital in managing PICS symptoms effectively.

We encourage you to ask questions, voice concerns, and seek support during your recovery process. Together, we can ensure the best possible outcomes for patients leaving the ICU.

Stay strong as you journey towards wellness. If you have any further questions about PICS or other aspects of your care, please don't hesitate to reach out.

Sincerely,
[Your Name]
[Your Title]
[Your Healthcare Organization]"

**Text 8**

**mistral_Prompt1_1**

Title: Understanding Post-Intensive Care Syndrome (PICS)

Dear Valued Patients and Families,

We are committed to providing you with comprehensive information about your health journey, including terms and conditions that may arise after a critical illness or prolonged stay in an intensive care unit. Today, we will discuss Post-Intensive Care Syndrome (PICS).

Post-Intensive Care Syndrome is a term used to describe a group of physical, psychological, and cognitive problems some people experience after surviving a critical illness or extended stay in the Intensive Care Unit (ICU). PICS can affect various aspects of daily life, such as mobility, emotional wellbeing, and thinking abilities.

Here's an overview of each type of PICS:

1. Physical PICS: This refers to ongoing physical problems that may include weakness, fatigue, pain, or changes in appetite or sleep patterns. These symptoms can persist for weeks, months, or even years after leaving the ICU.

2. Psychological PICS: Survivors of critical illnesses may experience emotional challenges such as anxiety, depression, post-traumatic stress disorder (PTSD), and adjustment disorders. It's essential to seek support from healthcare professionals if you notice changes in your emotional wellbeing after leaving the ICU.

3. Cognitive PICS: Some survivors may have difficulties with memory, concentration, or problem-solving abilities. This type of PICS is often referred to as "brain fog" and can significantly impact daily activities like work or managing personal finances.

PICS can affect anyone who has spent time in the ICU, regardless of age or severity of illness. It's essential to be aware of these potential complications so you can take steps towards recovery and rehabilitation with your healthcare team.

Remember, recovering from a critical illness is not just about getting better physically; it also involves addressing any psychological, cognitive, or emotional challenges that may arise. Open communication with your healthcare providers and loved ones is crucial in managing PICS symptoms effectively.

We encourage you to ask questions, voice concerns, and seek support during your recovery process. Together, we can ensure the best possible outcomes for patients leaving the ICU.

Stay strong and take care of yourself as you journey towards wellness.

Sincerely,
[Your Name]
[Your Title]
[Your Healthcare Organization]

**Text 9**

**mistral_Prompt4_3**

Search Phase:
In our search for reliable information on Post-Intensive Care Syndrome (PICS), we found several studies and guidelines. The Society of Critical Care Medicine (SCCM) in the United States proposed PICS in 2012 to address long-term problems affecting critically ill patients, such as those with sepsis, after their intensive care unit (ICU) stay. These issues can include physical, cognitive, and mental health disorders.

Generation Phase:
Post-Intensive Care Syndrome (PICS) is a group of problems that may occur during or after an ICU stay, affecting not only the long-term prognosis of critically ill patients but also their families' mental health. These issues can include physical weakness, difficulty thinking clearly, and emotional distress such as anxiety and depression.

Causes:
PICS is often due to the intense treatments required in the ICU, such as mechanical ventilation, sedatives, and immobilization. These interventions can lead to muscle weakness, cognitive impairments, and psychological distress. In addition, the critical illness itself can cause damage to various organs, which may contribute to PICS symptoms.

Symptoms:
Physical symptoms of PICS may include muscle weakness, fatigue, difficulty walking or climbing stairs, and changes in appetite or sleep patterns. Cognitive symptoms might be problems with memory, concentration, and the ability to make decisions. Emotional symptoms could manifest as anxiety, depression, post-traumatic stress disorder (PTSD), or adjustment difficulties.

Treatment:
Treatment for PICS typically involves a combination of physical therapy, cognitive rehabilitation, and psychological counseling. Physical therapy can help improve muscle strength and mobility, while cognitive rehabilitation can assist with memory and thinking skills. Psychological counseling can address emotional concerns such as anxiety, depression, or PTSD.

Daily Life Advice:
- Take it easy and rest when needed, but try to stay active as much as possible to help regain strength and mobility.
- Focus on a healthy diet and good sleep habits to support recovery.
- Seek out cognitive rehabilitation programs if you're having trouble with memory or thinking skills.
- Reach out for emotional support from friends, family, or mental health professionals if needed.
- Be open about your feelings and experiences with healthcare providers to ensure that they can address any concerns effectively.
