# Supplementary material for: Use of Commercially Available Large Language Models to Generate Information Leaflets on Post–Intensive Care Syndrome: Clinical Utility Assessment
Source: JMIR Form Res. 2026 May 14;10:e81606. doi: 10.2196/81606 (PMC13175452; doi:10.2196/81606)
Supplement: Multimedia Appendix 5 [file formative-v10-e81606-s005.docx]

**Detailed Screening and Expert Selection Process**

**Detailed Screening Process**

**Step 1: Initial Screening (72 → 53 Texts)**

A total of 72 texts were generated using six LLMs across four prompt designs, with three independent outputs per model–prompt combination (6 × 4 × 3).

Sixteen texts containing fewer than 500 Japanese characters and three texts that did not mention PICS were excluded, with no overlap between the two criteria.

As a result, 53 texts remained for further screening.

Details are provided in Appendix 2, where “Yes” indicates that the exclusion criterion was met.

**Step 2: Selection of Representative Texts per Model–Prompt Combination (53 → 21 Texts)**

From the remaining 53 texts, one representative output was selected for each LLM–prompt combination.

Specifically, the longest text was chosen within each combination. In cases where multiple outputs had identical character counts, the earliest generated output was selected.

This process resulted in 21 texts.

**Program Error Note**

For MedLLaMA Prompt 2, the longest output (Prompt2_2, 693 characters) should have been selected. However, Prompt2_3 (614 characters) was inadvertently selected due to a program error.

**Step 3: Model-Level Balancing Across Prompts (21 → 16 Texts)**

To ensure balanced representation across prompts and models, we adjusted the selection so that for each prompt, one text from each selected LLM was included.

GPT-4, Llama3:7b, and Mistral already had one representative text per prompt and were retained without modification.In contrast, Meditron, MedLLaMA, and Gemma had only three remaining texts each and did not cover all prompts. At the time of the study, MedLLaMA ranked higher and demonstrated greater stability than Meditron in publicly available benchmarks (e.g., Hugging Face rankings). Gemma was designed as a lightweight model and was ranked below GPT-4. Therefore, prioritizing **model-level representativeness**, MedLLaMA was selected among these three candidates. For Prompt 3, all MedLLaMA outputs were under 500 characters and had been excluded in Step 1. To maintain model–prompt balance, the longest of these outputs (Prompt3_3, 386 characters) was reinstated.

Ultimately, four models (GPT-4, Llama3:7b, MedLLaMA, and Mistral) across four prompts were selected, resulting in 16 texts (4 models × 4 prompts). The final 16 texts are listed in **Appendix 3**.

**Step 4: Expert Screening (16 → 9 Texts)**

The 16 selected texts were translated into Japanese and formatted as PDF files for expert review.

Two board-certified physicians independently evaluated the texts for suitability as patient- and family-facing materials.

Based on this expert screening process, 9 texts were ultimately retained (see **Appendix 4**).

One text (GPT4_Prompt3_1) was unintentionally omitted during the PDF conversion process and was therefore not evaluated, resulting in its exclusion.

**Expert Selection Process: From 16 to 9 Candidate Texts**

Two board-certified physicians (Physician A and Physician B) independently screened each candidate text for suitability as patient- and family-facing educational material. When their initial assessments differed, the texts were reassessed through discussion, and a final inclusion or exclusion decision was made based on overall appropriateness. Text ③ was not evaluated due to an unexpected omission.

**① GPT4_Prompt1_1**

**Initial assessment:** Acceptable by both evaluators.

**Final decision:** Accepted.**② GPT4_Prompt2_1**

**Initial assessment:**

Physician A: Not acceptable (healthcare-professional–oriented tone).

Physician B: Borderline (conclusion section inappropriate for families).

**Reassessment:** Despite the professional tone, the content itself was considered reasonable.
**Final decision:** Accepted.**③ GPT4_Prompt3_1**

Not evaluated due to unexpected omission.**④ GPT4_Prompt4_1**

**Initial assessment:**

Physician A: Acceptable.

Physician B: Borderline due to medical terminology in the explanation of PICS causes.

**Reassessment:** The use of medical terminology was considered acceptable overall.

**Final decision:** Accepted.**⑤ Llama3:7b_Prompt1_1**

**Initial assessment:** Acceptable by both evaluators.

**Final decision:** Accepted.**⑥ Llama3:7b_Prompt2_2**

**Initial assessment:** Not acceptable by both evaluators (healthcare-professional orientation; unclear format; insufficient content).

**Final decision:** Excluded**⑦ Llama3:7b_Prompt3_3**

**Initial assessment:** Acceptable by both evaluators.

**Final decision:** Accepted.**⑧ Llama3:7b_Prompt4_1**

**Initial assessment:** Acceptable by both evaluators.

**Final decision:** Accepted.**⑨ Medllama_Prompt1_3**

**Initial assessment:**

Physician A: Borderline (concern regarding encouragement phrasing).

Physician B: Acceptable.

**Reassessment:** Minor wording concerns were noted but not considered exclusionary.

**Final decision:** Accepted.**⑩ Medllama_Prompt2_3**

**Initial assessment:** Not acceptable by both evaluators (insufficient information; healthcare-professional orientation).

**Final decision:** Excluded.**⑪ Medllama_Prompt3_3**

**Initial assessment:**

Physician A: Borderline (limited information).

Physician B: Not acceptable (insufficient content).

**Reassessment:** Determined to lack sufficient content for meaningful evaluation.

**Final decision:** Excluded.

**⑫ Medllama_Prompt4_3**

**Initial assessment:** Not acceptable by both evaluators (limited information; inclusion of unnecessary or healthcare-oriented phrasing).

**Final decision:** Excluded.

**⑬ mistral_Prompt1_1**

**Initial assessment:**

Physician A: Borderline (unnatural Japanese expressions).

Physician B: Borderline (overly formal tone).

**Reassessment:** Although stylistic concerns remained, the overall content was deemed suitable for retention.

**Final decision:** Accepted. **⑭ mistral_Prompt2_3**

**Initial assessment:** Not acceptable by both evaluators (directed toward healthcare professionals; insufficient content).

**Final decision:** Excluded.**⑮ mistral_Prompt3_2**

**Initial assessment:**

Physician A: Acceptable.

Physician B: Borderline (confusing question order; somewhat limited content).

**Reassessment:** The structure was considered inappropriate for patient- and family-facing materials.

**Final decision:** Excluded.**⑯ mistral_Prompt4_3**

**Initial assessment:**

Physician A: Not acceptable (resembles explanation of search results).

Physician B: Borderline (references to “search phases” and “generation phases”).

**Reassessment:** Despite stylistic concerns, the content itself was considered acceptable.
**Final decision:** Accepted.

**Final Selection**

**Accepted for further analysis:** ①, ②, ④, ⑤, ⑦, ⑧, ⑨, ⑬, ⑯

**Excluded:** ⑥, ⑩, ⑪, ⑫, ⑭, ⑮

**Not evaluated:** ③
