# Supplementary material for: Use of Commercially Available Large Language Models to Generate Information Leaflets on Post–Intensive Care Syndrome: Clinical Utility Assessment
Source: JMIR Form Res. 2026 May 14;10:e81606. doi: 10.2196/81606 (PMC13175452; doi:10.2196/81606)
Supplement: Multimedia Appendix 11 [file formative-v10-e81606-s011.docx]

**Outcome: Average score**

| **Variable** | **β (SE)** | **95% CI** | **p value** |
| --- | --- | --- | --- |
| **Intercept** | 9.92 (0.27) | 9.39 to 10.46 | <.001 |
| **Large language model (reference: llama3:70b)** | | | |
| ChatGPT-4o | 0.08 (0.23) | −0.36 to 0.53 | .715 |
| Gemma | −1.73 (0.43) | −2.57 to −0.88 | <.001 |
| Medllama | −0.62 (0.27) | −1.15 to −0.09 | .022 |
| meditrone:7b | −2.63 (0.38) | −3.37 to −1.89 | <.001 |
| mistral | 0.04 (0.21) | −0.37 to 0.45 | .841 |
| **Prompt (reference: Zero-shot)** | | | |
| Few-shot | −0.19 (0.34) | −0.85 to 0.47 | .568 |
| Step-by-step | 0.33 (0.23) | −0.13 to 0.79 | .154 |
| **Text-augmented prompting approach (reference: without context)** | | | |
| With context | −0.72 (0.27) | −1.25 to −0.19 | .007 |
| **Output number (reference: 1st)** | | | |
| 2nd | −0.01 (0.24) | −0.48 to 0.46 | .970 |
| 3rd | −0.36 (0.27) | −0.89 to 0.17 | .185 |

SE: standard error; CI: confidence interval.
