# Supplementary material for: Use of Commercially Available Large Language Models to Generate Information Leaflets on Post–Intensive Care Syndrome: Clinical Utility Assessment
Source: JMIR Form Res. 2026 May 14;10:e81606. doi: 10.2196/81606 (PMC13175452; doi:10.2196/81606)
Supplement: Multimedia Appendix 12 [file formative-v10-e81606-s012.docx]

**Outcome: Average score**

| **Variable** | **β (SE)** | **95% CI** | **p value** |
| --- | --- | --- | --- |
| **Intercept** | 9.92 (0.27) | 9.40 to 10.44 | <.001 |
| **Large language model (reference: llama3:70b)** | | | |
| ChatGPT-4o | −0.03 (0.17) | −0.36 to 0.30 | .869 |
| Gemma | −1.87 (0.41) | −2.67 to −1.07 | <.001 |
| Medllama | −0.80 (0.23) | −1.25 to −0.36 | <.001 |
| meditrone:7b | −2.46 (0.45) | −3.35 to −1.57 | <.001 |
| mistral | 0.04 (0.17) | −0.29 to 0.38 | .801 |
| **Prompt (reference: Zero-shot)** | | | |
| Few-shot | −0.23 (0.36) | −0.93 to 0.48 | .532 |
| Step-by-step | 0.27 (0.20) | −0.13 to 0.67 | .186 |
| **Text-augmented prompting approach (reference: without context)** | | | |
| With context | −0.66 (0.26) | −1.18 to −0.14 | .012 |
| **Output number (reference: 1st)** | | | |
| 2nd | −0.03 (0.26) | −0.55 to 0.49 | .916 |
| 3rd | −0.25 (0.27) | −0.78 to 0.27 | .340 |

SE: standard error; CI: confidence interval.
