# Supplementary material for: Use of Commercially Available Large Language Models to Generate Information Leaflets on Post–Intensive Care Syndrome: Clinical Utility Assessment
Source: JMIR Form Res. 2026 May 14;10:e81606. doi: 10.2196/81606 (PMC13175452; doi:10.2196/81606)
Supplement: Multimedia Appendix 13 [file formative-v10-e81606-s013.docx]

**Outcome: Average score**

| **Variable** | **β (SE)** | **95% CI** | **p value** |
| --- | --- | --- | --- |
| **Intercept** | 9.80 (0.42) | 8.99 to 10.61 | <.001 |
| **Large language model (reference: llama3:70b)** | | | |
| ChatGPT-4o | 0.08 (0.38) | −0.66 to 0.83 | .827 |
| Gemma | −1.73 (0.76) | −3.22 to −0.23 | .024 |
| Medllama | −0.62 (0.32) | −1.24 to 0.00 | .052 |
| meditrone:7b | −2.63 (0.33) | −3.28 to −1.97 | <.001 |
| mistral | 0.04 (0.34) | −0.63 to 0.71 | .903 |
| **Prompt (reference: Zero-shot)** | | | |
| Few-shot | −0.19 (0.41) | −1.00 to 0.61 | .639 |
| Step-by-step | 0.33 (0.25) | −0.15 to 0.82 | .173 |
| **Text-augmented prompting approach (reference: without context)** | | | |
| With context | −0.72 (0.44) | −1.58 to 0.13 | .096 |

SE: standard error; CI: confidence interval.
