# Supplementary material for: Use of Commercially Available Large Language Models to Generate Information Leaflets on Post–Intensive Care Syndrome: Clinical Utility Assessment
Source: JMIR Form Res. 2026 May 14;10:e81606. doi: 10.2196/81606 (PMC13175452; doi:10.2196/81606)
Supplement: Multimedia Appendix 14 [file formative-v10-e81606-s014.docx]

**Outcome: Average score**

| **Variable** | **β (SE)** | **95% CI** | **p value** |
| --- | --- | --- | --- |
| **Intercept** | 9.82 (0.42) | 8.99 to 10.65 | <.001 |
| **Large language model (reference: llama3:70b)** | | | |
| ChatGPT-4o | −0.03 (0.29) | −0.60 to 0.54 | .924 |
| Gemma | −1.87 (0.79) | −3.42 to −0.32 | .018 |
| Medllama | −0.80 (0.27) | −1.33 to −0.28 | .003 |
| meditrone:7b | −2.46 (0.44) | −3.33 to −1.60 | <.001 |
| mistral | 0.04 (0.31) | −0.56 to 0.64 | .888 |
| **Prompt (reference: Zero-shot)** | | | |
| Few-shot | −0.23 (0.41) | −1.03 to 0.58 | .582 |
| Step-by-step | 0.27 (0.24) | −0.20 to 0.74 | .259 |
| **Text-augmented prompting approach (reference: without context)** | | | |
| With context | −0.66 (0.48) | −1.60 to 0.28 | .166 |

SE: standard error; CI: confidence interval.
